# PAX6 upstream antisense RNA (PAUPAR) inhibits colorectal cancer progression through modulation of the microRNA (miR)-17-5p / zinc finger protein 750 (ZNF750) axis

**DOI:** 10.1080/21655979.2021.1940071

**Published:** 2021-07-21

**Authors:** Ruhui Wen, Chao Chen, Xiaohua Zhong, Chen Hu

**Affiliations:** Department of Gastrointestinal Surgery, Huizhou Municipal Central Hospital, Huizhou, Guangdong, China

**Keywords:** CRC, miR-17-5p, PAUPAR, ZNF750

## Abstract

Researchers have demonstrated that long non-coding RNAs (lncRNAs) are vital in colorectal cancer (CRC) progression. Here, we aimed to explore the function of lncRNA PAX6 upstream antisense RNA (PAUPAR) in the development of CRC. In the present study, PAUPAR and microRNA (miR)-17-5p expression levels in CRC tissues and cells were examined using quantitative real-time polymerase chain reaction (qRT-PCR). Western blot analysis was adopted to examine ZNF750 expression at the protein level in CRC cells. CRC cell proliferation was examined by colony formation experiment and 5-Bromo-2-deoxyUridine (BrdU) experiment. CRC cell migration and invasion were assessed by Transwell experiments. Apoptosis was measured using the TUNEL experiment. The targeting relationship between PAUPAR and miR-17-5p was confirmed using dual-luciferase reporter gene and RNA immunoprecipitation (RIP) experiments. We demonstrated that PAUPAR was markedly down-modulated in CRC, and its low expression was significantly related to increased T stage and local lymph node metastasis. Knockdown of PAUPAR enhanced CRC cell proliferation, migration and invasion, and restrained apoptosis relative to controls, whereas PAUPAR overexpression caused the opposite effects. Moreover, rescue experiments showed that miR-17-5p inhibitor could reverse the role of PAUPAR knockdown on the malignant phenotypes of CRC cells. Additionally, PAUPAR could positively regulate the expression of ZNF750 via repressing miR-17-5p. Taken together, these findings suggest that PAUPAR/miR-17-5p/ZNF750 axis is a novel mechanism implicated in CRC progression.

## Graphical abstractPAUPAR repressed the proliferation, migration and invasion of CRC cells, and promoted the apoptosis of CRC cells. PAUPAR sponged miR-17-5p and repressed its expression, and ZNF750 was demonstrated to be negatively regulated by miR-17-5p. PAUPAR could upregulate the expression of ZNF750 via downregulating miR-17-5p, and the functions of PAUPAR were dependent on its regulatory function on miR-17-5p/ZNF750 axis

**Figure:**
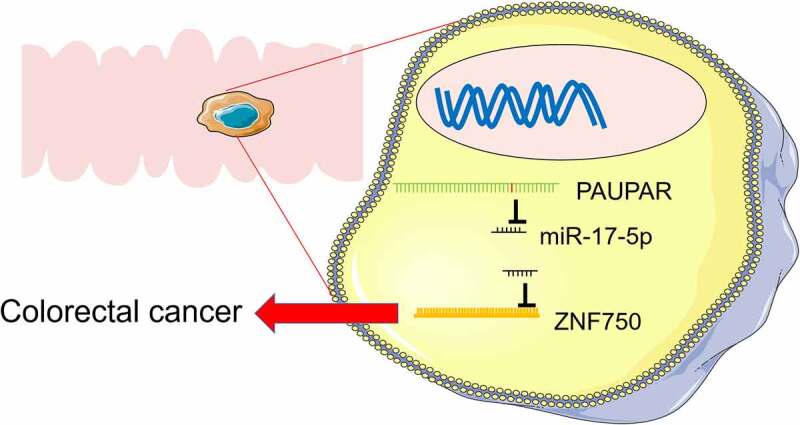

## Introduction

Colorectal cancer (CRC) is the third most commonly diagnosed malignancy and the second leading cause of cancer-related death worldwide [1]. There are more than 1.7 million newly diagnosed CRC cases and more than 800,000 deaths in 2018 [2]. Great progress has been made in diagnosis and therapy; however, the five-year survival rate of CRC patients with distant metastases is only 10–15% [3,4]. Hence, exploring potential molecular mechanisms of CRC progression promises to provide new therapeutic options for CRC patients.

Long non-coding RNAs (lncRNAs) are transcripts of more than 200 nucleotides in length without protein-coding function [5]. LncRNAs were initially regarded as transcriptional ‘noise,’ but they gained significant attention in recent years due to their regulatory functions in biological processes [6–8]. In recent years, accumulating evidence has indicated that the aberrant expression of lncRNAs is closely linked to diverse malignancies, including CRC [9–15]. For instance, lncRNA RP11 is overexpressed in CRC tissues and increases with CRC stage in patients; RP11 enhances migration, invasion, and epithelial–mesenchymal transition (EMT) of CRC cells and accelerates hepatic metastasis *in vivo* [11]. In addition, lncRNA GLCC1 enhances CRC carcinogenesis by directly interacting with the HSP90 chaperone to stabilize c-Myc from ubiquitination [12]. PAX6 upstream antisense RNA (PAUPAR) has been reported to inhibit the progression of several malignancies, including neuroblastoma and uveal melanoma [14,15]. Nonetheless, the function and regulatory mechanisms of PAUPAR in CRC are undefined.

MicroRNAs (miRNAs) are endogenous, small non-coding RNAs containing 18–25 nucleotides, which are highly conserved, time-specific, and tissue-specific [16]. MiRNAs modulate protein synthesis in the cytoplasm through base pairing with the 3ʹ untranslated regions (3ʹUTR) of target messenger RNAs (mRNAs) [17]. For instance, miR-6716-5p facilitates CRC metastasis via down-modulating NAT10 expression [18]. MiR-889 accelerates CRC progression via specifically repressing DAB2IP [19]. Furthermore, it has been demonstrated that miR-17-5p expression is markedly up-modulated in CRC tissues, indicating that miR-17-5p is cancer-promoting in CRC progression [20,21]. Nevertheless, the upstream mechanisms causing miR-17-5p overexpression remain unclear.

The gene of zinc finger protein 750 (ZNF750) is located at 17q25.3, and the protein mainly exists in stratified squamous epithelium. As a transcription factor, it participates in the terminal differentiation of epithelium. It can impede and enhance transcription of proliferation and differentiation-related genes, respectively [22,23]. It is reported that ZNF750 represses nasopharyngeal carcinoma development and progression by modulating the fibroblast growth factor 14 expression [22]. ZNF750 also suppresses the progression of other malignancies such as esophageal squamous cell carcinoma [23–25]. Importantly, a previous research confirms that ZNF750 exerts a tumor-suppressive effect in CRC [21]. Nevertheless, the complete upstream mechanism that causes the dysregulation of ZNF750 expression in CRC is not elucidated.

This work aims to explore the expression characteristics, biological function, and underlying mechanism of PAUPAR in CRC. Through the analysis of the ENCORI online website (http://starbase.sysu.edu.cn/), we found that there were predicted binding sites between PAUPAR and miR-17-5p. It was hypothesized that PAUPAR was down-regulated in CRC, and it inhibited CRC cell proliferation, migration, invasion and EMT via modulating miR-17-5p/ZNF750 axis,

## Materials and methods

### Samples collection

Forty-seven subjects with newly diagnosed CRC were selected. Cancerous tissues and paired paracancerous tissues (at >1 cm from the edge of the primary tumor) were obtained and immediately snap-frozen in liquid nitrogen. None of the patients underwent radiotherapy, chemotherapy, or targeted therapy before the surgery. The subjects were aware of the purpose of the study and signed written informed consent prior to enrollment. The research was conducted under the approval and guidance of the Ethics Committee of HuiZhou Municipal Hospital and in strict adherence to the Declaration of Helsinki.

### Cell culture and transfection

Human CRC cell lines (HCT116, HT29, LoVo, SW620, SW480, and HCT8), and human normal colonic epithelial cell line CCD841CoN were available from the American Type Culture Collection (ATCC, Manassas, VA, USA). All cells were maintained in the Dulbecco modified Eagle’s medium (DMEM) containing 10% fetal bovine serum (FBS, Tianhang Biotechnology Co. Ltd., Hangzhou, China), 100 U/mL penicillin, and 0.1 mg/mL streptomycin (Gibco, Grand Island, NY, USA) in a humidified incubator at 37°C with 5% CO_2_. The subculture was conducted using 0.25% trypsin (Ameresco, Framingham, MA, USA). The pcDNA3.1 vector carrying PAUPAR sequence and empty plasmid, small interfering RNA (siRNA) oligonucleotides targeting PAUPAR (si-PAUPAR), miR-17-5p mimics, and miR-17-5p inhibitors were designed and provided by Genechem Co., Ltd. (Shanghai, China). CRC cells were transfected with the vectors/oligonucleotides at a final concentration of 50 nM using Lipofectamine® 3000 (Invitrogen, Carlsbad, CA, USA) in accordance with the manufacturer’s instruction.

### Quantitative real-time PCR (qRT-PCR) and miRNA RT-PCR

Total RNA was extracted from tissues and cells using TRIzol reagent (Invitrogen, Carlsbad, CA, USA) under the guidance of the protocols. Total RNA was reverse transcribed using the first-strand cDNA system (Invitrogen, Grand Island, NY, USA). qRT-PCR was performed using the Light Cycler Fast Start DNA MasterPlus SYBR Green I kit (Roche Diagnostics, Burgess Hill, UK). GAPDH was used as the endogenous control. For miR-17-5p expression detection, reverse transcription was performed following Applied Biosystems TaqMan MicroRNA Assay kit (ThermoFisher, Carlsbad, CA, USA), and U6 was used as the endogenous control. Primers were procured from Genewiz (Suzhou, China), and the sequences are described in Table 1.

**Table 1.** Sequences used for qRT-PCR

PAUPAR	Forward	5ʹ-CTCGAACTCTCTTCCTTGCCAGA-3’
	Reverse	5ʹ-CGCTGCAAATAGATGGGTGAGTG-3’
ZNF750	Forward	5ʹ-TACAGCCCCAGGAACATC-3’
	Reverse	5ʹ-GCTCCTTGCTGGGATTTT-3’
GAPDH	Forward	5ʹ-ＡAGGTCGGTGTGAACGGATTTG-3’
	Reverse	5ʹ-AGCACTGTGTTGGCGTACAG-3’
miR-17-5p	Forward	5ʹ-CTACCTGCACTGTAAGCACTTTG-3’
	Reverse	Uni-miR qRT-PCR primer
U6	Forward	5ʹ-GAGGCACAGCGGAACG-3’
	Reverse	5ʹ-CTACCACATAGTCCAGG-3’

### Colony formation experiment

Cells were inoculated in 6-well plates at a total of 1000 cells/well for 2 weeks, and fresh medium was replaced every 3 days. After 14 days, the supernatant was discarded and the cells were rinsed twice with 1 ml of phosphate buffered saline (PBS). The colonies were fixed with 4% paraformaldehyde for 10 min and then carefully rinsed 3 times with PBS. Subsequently, 0.1% crystal violet solution was added to each well, and the cells were stained for 15 min. After washing, the colonies were observed and counted.

### BrdU experiment

CRC cells cultured in 12-well plates were labeled with 10 μmol/L BrdU (BD Pharmingen, San Diego, CA, USA) for 30 min. Cells were fixed with 1 mL of Carnoy’s fixative. Subsequently, the DNA was denatured in 2 M hydrochloric acid at 37°C for 60 min and then washed three times in borate buffer (0.1 M, pH = 8.5). After incubation in blocking buffer, the cells were incubated with 100 μL of anti-BrdU antibody (ab8152, 1:300, Abcam, Cambridge, UK) overnight at 4°C. Subsequently, the cells were incubated with Texas Red-labeled anti-mouse goat IgG for 30 min. After the nuclei were stained with DAPI staining solution, the cells were observed under a fluorescence microscope. The number of BrdU-positive cells was counted and averaged.

### Transwell migration and invasion experiments

Invasion experiment: Transwell chambers pre-coated with Matrigel (BD Biosciences, Franklin Lakes, NJ, USA) were used. Briefly, 500 μL of medium containing 10% serum was added to the lower compartment and 200 μL of serum-free medium containing 5 × 10^4^ cells was added to the upper compartment, and the cells were cultured. 48 h later, the upper chamber was removed, the cells remaining on the upper surface of the membrane were wiped off, and cells which passed through the membrane were stained using 0.1% crystal violet and fixed in methanol for 30 min. After rinsing with PBS solution, the stained cells were observed under an inverted microscope (Leica, Wetzlar, Germany), photographed, and counted. In the migration experiment, the same steps as in the invasion experiment were used except that Matrigel was not added.

### Apoptosis experiments

Briefly, CRC cells were cultured on cover slips, which were placed in 6-well plate. Subsequently, the medium was discarded, and the cells were fixed with fixative for 15 min, and then the cells were blocked with blocking solution at room temperature for 15 min. Next, the cells were incubated with 0.1% Triton X-100 solution, and then incubated with a TUNEL BrightGreen Apoptosis Detection Kit (Vazyme, Nanjing, China) at 37°C for 1 h. Next, the cells were incubated with DAPI staining solution for 5 min in the dark. Subsequently, the cells were washed by PBS, and then observed under a fluorescence microscope.

### Western blot

RIPA lysis buffer (Beyotime, Shanghai, China) was used to lyse the CRC cells, and the lysate was collected for total protein extraction after centrifugation (12,000 r/min, 30 min). The protein concentration was determined using a BCA kit (Invitrogen, Shanghai, China). The loading volume was adjusted according to the protein concentration, and the protein (20 μg/lane) was transferred to polyvinylidene difluoride (PVDF) membranes (Millipore, Billerica, MA, USA) after sodium dodecyl sulfate-polyacrylamide gel (SDS-PAGE). Antigen blocking was performed in 5% skim milk, and then the primary antibody was added to incubate the PVDF membranes overnight at 4°C. After rinsing the membrane, a secondary antibody was added to incubate the PVDF membrane for 1 h at 37°C. After the membranes were washed again, an enhanced chemiluminescence (ECL) kit (Biosci, Wuhan, China) was used to develop the protein bands. GAPDH was employed as an internal reference. The primary antibodies involved in this research were as follows: anti-ZNF750 antibody (Abbkine, ABP60986), anti-E-cadherin antibody (Abcam, ab194982), anti-Vimentin antibody (Abcam, ab92547), and anti-GAPDH antibody (Abcam, ab8245).

### RIP experiment

The Magna RIP RNA-binding protein immunoprecipitation kit (Millipore, Billerica, MA, USA) was utilized for RIP experiments. HEK-293 cells were transfected with Ago2 plasmid or control vector. Then, 1 × 10^7^ cells were then suspended in 100 μL of RIP lysis buffer containing a protease inhibitor cocktail and an RNase inhibitor. About 200 μL of cell lysate and 5 μg rabbit Anti-Argonaute-2 antibody (Abcam, ab186733, 1:500) or anti-IgG antibody (Abcam, ab200699, 1:500), which was coupled with magnetic bead, were incubated overnight at 4°C with rotation. Next, the immunoprecipitate was harvested, and protein was removed by proteinase K. Ultimately, RNA was extracted and the enrichment of PAUPAR was measured by qRT-PCR.

### Luciferase reporter gene experiment

The reporter plasmids (pGL3-Firefly Luciferase-Renilla_Luciferase Reporter carrying the PAUPAR sequence or a mutant sequence) were designed by Genechem Co., Ltd. (Shanghai, China). The reporter plasmid and miR-17-5p mimic or negative control mimic (RiboBio, Guangzhou, China) were co-transfected into HEK-293 T cells and the cells were cultured for 48 h. The luciferase activity was then examined using the Dual-Luciferase Reporter Assay System (Promega, Madison, WI, USA).

### In vivo experiment

Female BALB/c nude mice (4 to 6 weeks old, Charles River Laboratories, Guangdong, China) were housed in a pathogen-free facility with a temperature of 24 ± 1°C, a humidity of 50%, 12-h light-dark cycle, and ad libitum access to water and food. They were divided into two groups (PAUPAR overexpression group *v.s*. NC group, n = 10 per group). Then, 1 × 10^7^ HCT8 cells were injected into each mouse via caudal vein, and 2 weeks later, the lung tissue was taken from the mice and fixed in 10% formalin. After paraffin-embedding, the tissues were sliced (thickness: 4 μm) and baked at 65°C for 20 min. The tissue sections were dewaxed and hydrated, and then hematoxylin-eosin staining was performed, and finally, the sections were observed under the microscope to evaluate the metastasis.

### Statistical analysis

All the experiments were performed in triplicate. Statistical analysis was executed using SPSS 17.0 statistical software (SPSS Inc., Chicago, IL, USA) and GraphPad Prism 8.0.1 software (GraphPad Software, Inc., La Jolla, CA, USA). The data were expressed as ‘mean ± standard deviation (x ± s).’ Kolmogorov–Smirnov test was used to determine the normality of the distribution of data in each group. *t*-tests were adopted to compare the means of the two groups, and one-way ANOVA was employed to compare the means of multiple groups. *P* < 0.05 indicated statistical significance.

## Results

This work was aimed to explore the expression pattern, biological function, and underlying mechanism of PAUPAR in CRC, and it was demonstrated thatPAUPAR expression was down-modulated in CRC tissues and cell lines. We also reported that PAUPAR could inhibit CRC cells’ malignant phenotype by sponging miR-17-5p and upregulating the expression of ZNF750.

### PAUPAR underexpression in CRC was linked to unfavorable clinical parameters

**Figure 1.**
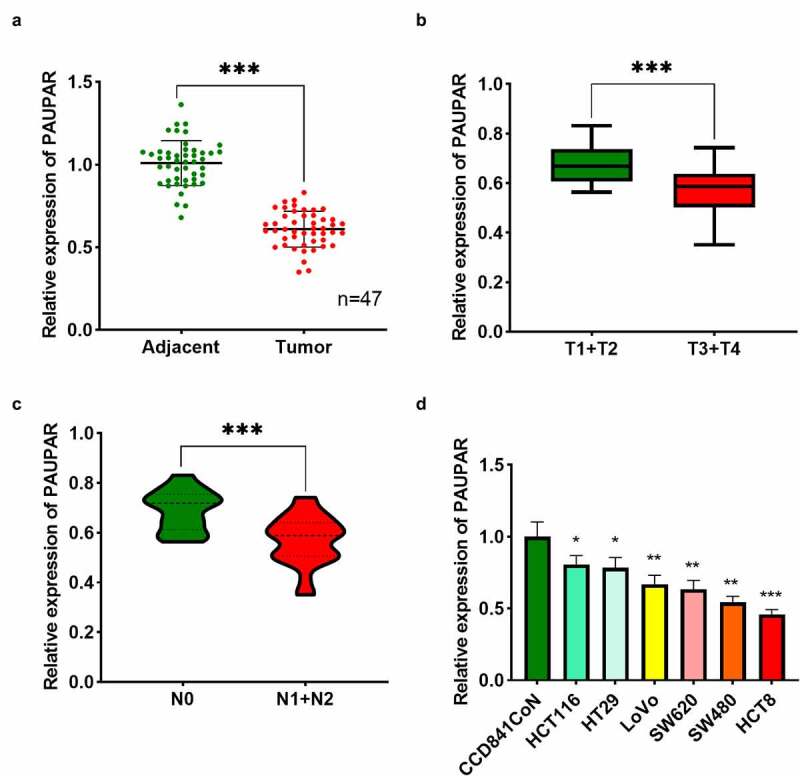
Low expression of PAUPAR in CRC correlated with poor clinical indicators

PAUPAR expression in tumor tissues and matched paracancerous tissues of 47 CRC subjects was examined using qRT-PCR. The findings revealed that PAUPAR expression was remarkably down-modulated in CRC tissues as opposed to adjacent non-tumor tissues (Figure 1(a)). Notably, PAUPAR low expression was strongly related to increased T stage (Figure 1(b)). CRC patients with lymph node metastasis had lower PAUPAR expression in the cancer tissues relative to patients without lymph node metastasis (Figure 1(c)). The data of qRT-PCR implied that PAUPAR expression was remarkably down-modulated in six different CRC cell lines (HCT116, HT29, LoVo, SW620, SW480, and HCT8) compared to CCD841CoN cells (Figure 1(d)). The findings indicated that PAUPAR was a tumor-suppressive factor in CRC.

(a) PAUPAR expression in human CRC tissues and paracancerous normal tissues was detected by qRT-PCR (n = 47). (b) The relationship between PAUPAR expression and T stage in CRC patients was analyzed. (c) The relationship between PAUPAR expression and lymph node metastasis in CRC patients was analyzed. (d) PAUPAR expression in normal colonic epithelial cells (CCD841 CoN cells) and six types of CRC cells (HCT116, HT29, LoVo, SW620, SW480, and HCT8) were determined by qRT-PCR. All the experiments were performed in triplicate. * *P* < 0.05, ** *P* < 0.01, *** *P* < 0.001.

### PAUPAR restrained CRC cell proliferation, migration and invasion, and enhanced apoptosis

**Figure 2.**
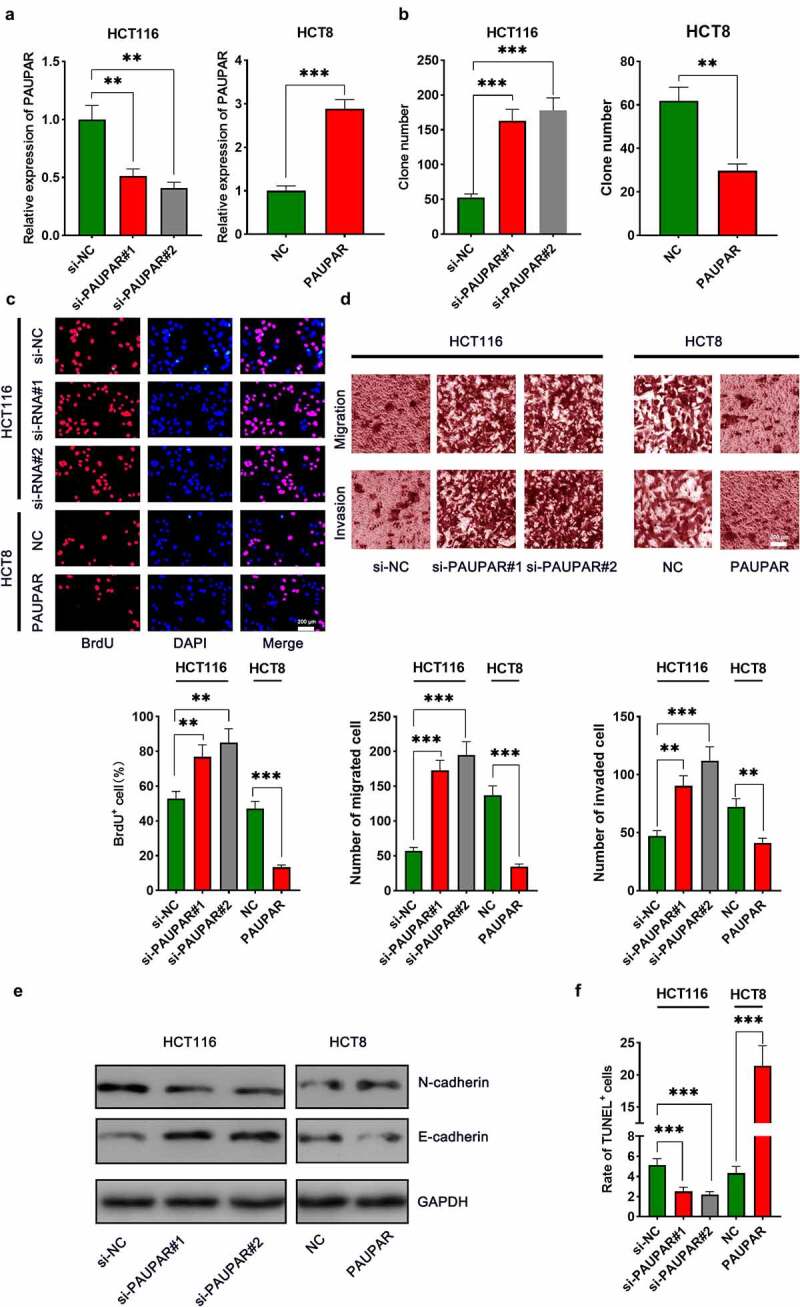
PAUPAR restricted proliferation, migration and invasion of CRC cells and promoted apoptosis

The aforementioned results suggested that among the six CRC cell lines, PAUPAR expression was the highest in HCT116 cells and the lowest in HCT8 cells. Thus, HCT8 cells were transfected with overexpressing PAUPAR plasmid and PAUPAR expression in HCT116 cells was knocked down by siRNA-mediated gene silencing. The overexpression and knockdown models were successfully constructed and validated by qRT-PCR (Figure 2(a)). Colony formation and BrdU experiments revealed that PAUPAR overexpression markedly repressed HCT8 cell proliferation relative to the control group (Figure 2(b,c)). Transwell experiments implied that PAUPAR overexpression markedly restrained the migration and invasion of HCT8 cells (Figure 2(d)). Additionally, EMT indicators, including E-cadherin and N-cadherin, were examined by Western blot. The data suggested that PAUPAR overexpression remarkably increased E-cadherin and decreased N-cadherin expression relative to the control group (Figure 2(e)). TUNEL experiments showed that the up-modulation of PAUPAR enhanced apoptosis as opposed to the control group (Figure 2(f)). Conversely, knocking down the PAUPAR in HCT116 cells exerted the opposite function (Figure 2(b–f)).

(a) HCT8 cells were transfected with PAUPAR overexpression plasmid, and the PAUPAR expression in HCT116 cells was knocked down by siRNA. Successful transfection was confirmed by qRT-PCR. (b,c) Colony formation assay and BrdU assay were used to detect cell proliferation. (d) Transwell experiments were used to detect cell migration and invasion. (e) Western blot was used to detect the expression of EMT-related markers, including E-cadherin and N-cadherin. (f) The apoptosis of CRC cells was detected by TUNEL assay. All the experiments were performed in triplicate. ** *P* < 0.01, *** *P* < 0.001.

### PAUPAR acted as a molecular sponge of miR-17-5p

**Figure 3.**
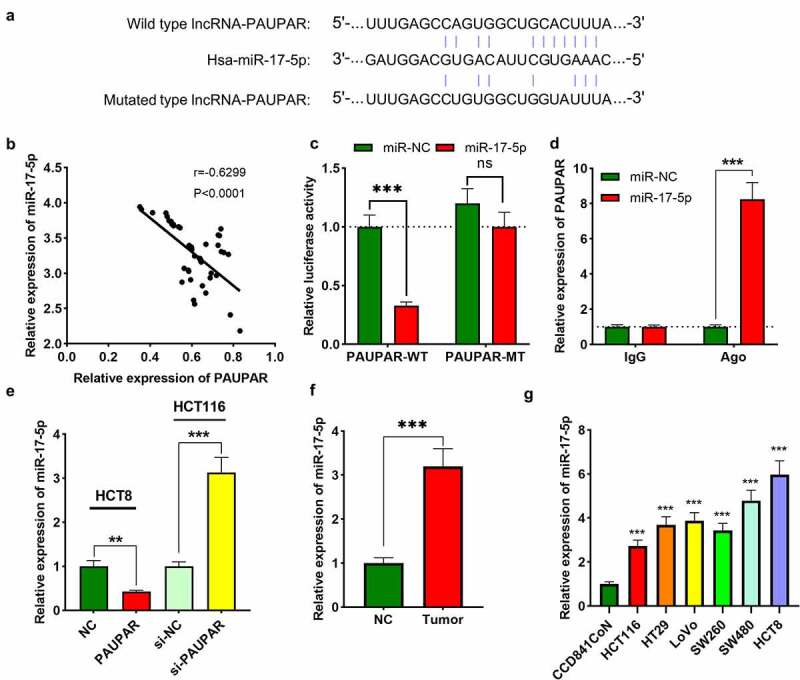
PAUPAR adsorbed miR-17-5p

To explore the mechanism by which PAUPAR affects the phenotypes of CRC cells, we performed bioinformatics analysis. ENCORI database (http://starbase.sysu.edu.cn/) indicated a potential binding site between PAUPAR and miR-17-5p (Figure 3(a)). In addition, their expression levels were found to be strongly negatively correlated in CRC cancer tissues (Figure 3(b)). Dual-luciferase reporter gene experiments confirmed that miR-17-5p remarkably repressed the luciferase activity of PAUPAR-WT reporter, whereas it did not remarkably alter the luciferase activity of PAUPAR-MT reporter (Figure 3(c)). Furthermore, RIP experiments confirmed that relative to the control IgG, PAUPAR and miR-17-5p were enriched in Ago2-containing microribonucleoproteins (Figure 3(d)). It was subsequently found that PAUPAR overexpression in HCT8 cells markedly suppressed miR-17-5p expression, whereas knocking down PAUPAR in HCT116 cells caused an increase in miR-17-5p expression (Figure 3(e)). Also, the data of qRT-PCR suggested that miR-17-5p expression was up-modulated in CRC tissues and cell lines in comparison with adjacent non-tumor tissues and cells (Figure 3(f,g)). Three online databases (miRDIP, TargetScan, and StarBase) were searched computationally for potential target genes that are complementary to miR-17-5p. A total of 142 candidate targets was predicted by these 3 tools (Supplementary Figure 1(a)). Then, KEGG pathway analysis and GO analysis were performed to elucidate the potential biological functions of miR-17-5p integrated-signature. Interestingly, the KEGG and GO pathways enriched for the miR-17-5p targets were mainly associated with cancer-related pathways, including the AMPK and WNT signaling pathways, etc. (Supplementary Figure 1(b,c)).

(a) Bioinformatics analysis indicated a potential binding site between PAUPAR and miR-17-5p. (b) Pearson’s analysis showed a negative correlation between PAUPAR and miR-17-5p expression levels in CRC tissues. (c) Dual-luciferase reporter gene assay showed that miR-17-5p inhibited the luciferase activity of PAUPAR-WT reporter, but not the PAUPAR-WT reporter. (d) RIP experiments were employed to determine the interaction between PAUPAR and miR-17-5p. (e) After up-regulating PAUPAR in HCT8 cells and knocking down PAUPAR in HCT116 cells, miR-17-5p expression was detected by qRT-PCR. (f) MiR-17-5p expression in cancerous tissues and adjacent non-tumor tissues were analyzed by qRT-PCR. (g) MiR-17-5p expression in normal colonic epithelial cells and CRC cell lines were determined by qRT-PCR. All the experiments were performed in triplicate. ** *P* < 0.01, *** *P* < 0.001, ns: no significance.

### MiR-17-5p enhanced malignant characteristics of CRC cells

**Figure 4.**
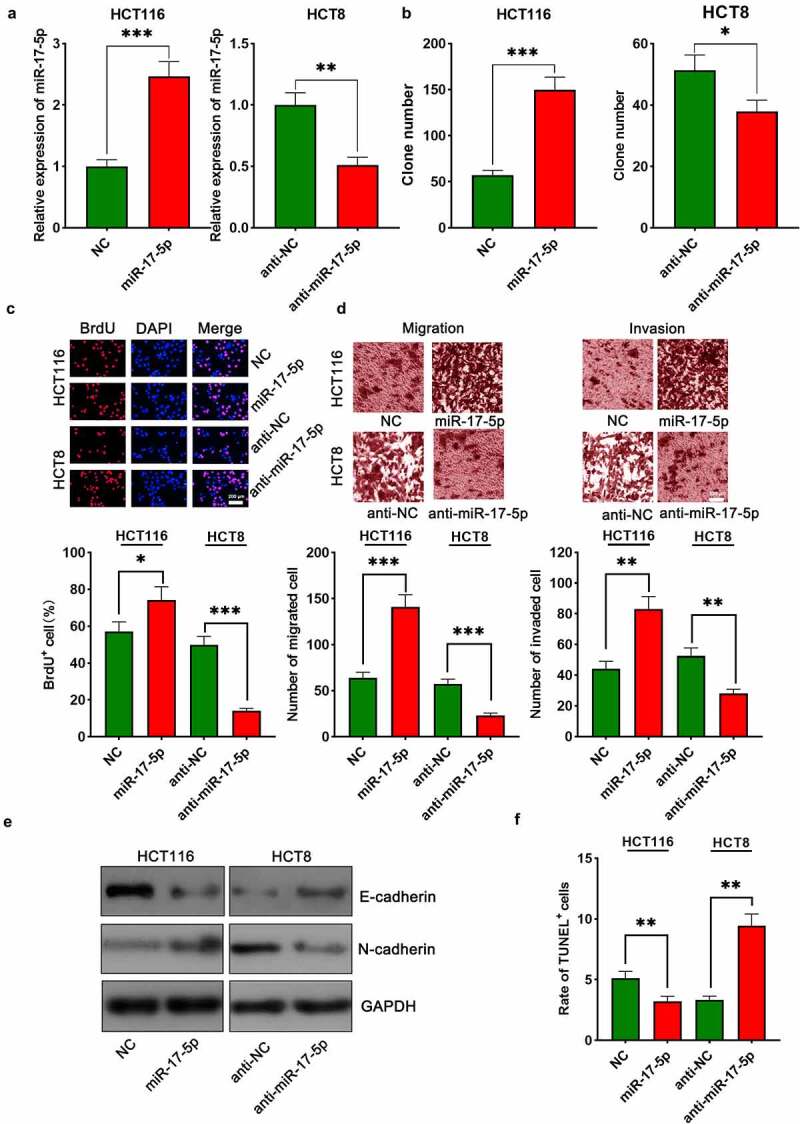
miR-17-5p played a pro-cancer role in CRC

To investigate the biological function of miR-17-5p in CRC, HCT116 cells were transfected with miR-17-5p mimics, and HCT8 cells were transfected with anti-miR-17-5p, and the successful transfection was verified by qRT-PCR (Figure 4(a)). The function of miR-17-5p in regulating the malignant biological behaviors of CRC cells was assessed by colony formation assay, BrdU assay, Transwell assay, TUNEL assay, and Western blot assay. Up-modulation of miR-17-5p was found to facilitate colony formation, migration, and invasion, EMT processes of CRC cells and restrained apoptosis (Figure 4(b–f), Supplementary Figure 2). On the contrary, transfection with anti-miR-17-5p markedly suppressed the above malignant biological behavior relative to the control group (Figure 4(b–f), Supplementary Figure 2).

(a) HCT116 cells were transfected with miR-17-5p mimics, and HCT8 cells were transfected with anti-miR-17-5p. qRT-PCR was used to detect transfection efficiency. (b,c) Colony formation assay and BrdU assay were used to detect cell proliferation. (d) Transwell experiments were used to detect cell migration and invasion. (e) Western blot was used to detect the expression of EMT-related markers, including E-cadherin and N-cadherin. (f) The apoptosis of CRC cells was detected by TUNEL assay. All the experiments were performed in triplicate. * *P* < 0.05, ** *P* < 0.01, *** *P* < 0.001.

### PAUPAR participated in modulating CRC cell proliferation and metastasis by sponging miR-17-5p

**Figure 5.**
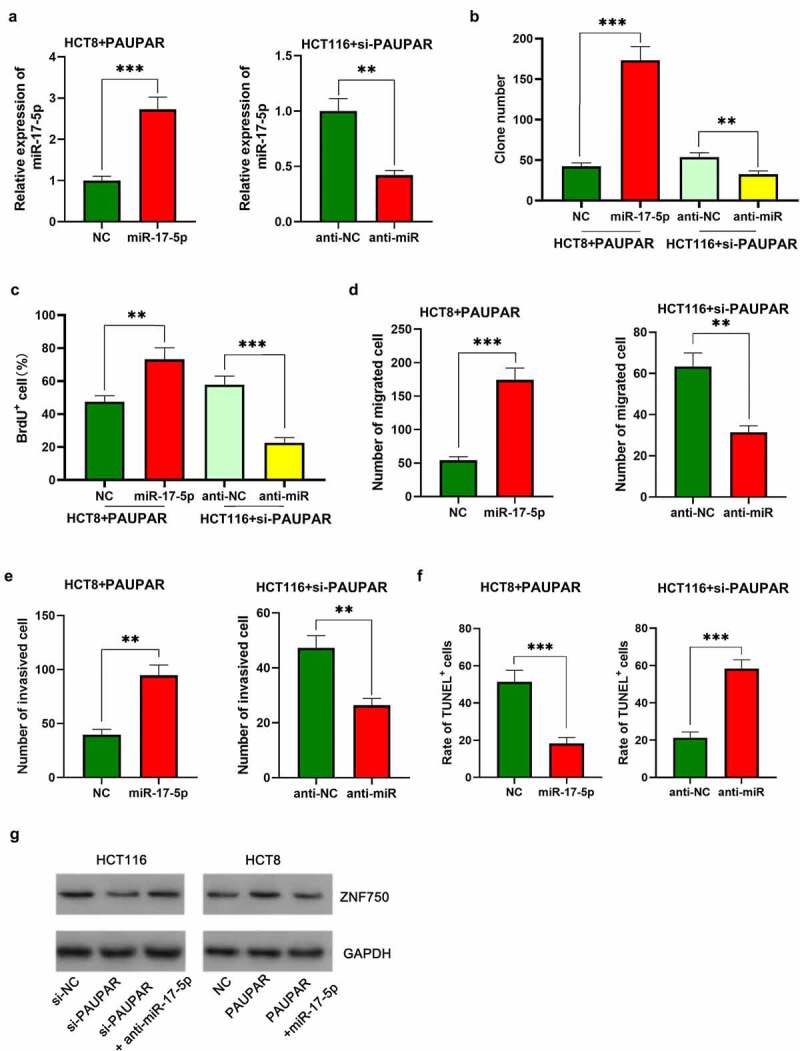
Restoration of miR-17-5p expression reversed the inhibitory effect of PAUPAR on the malignant phenotypes of CRC cells

To elaborate on the function of the PAUPAR/miR-17-5p axis in CRC, miR-17-5p mimics were transfected into HCT8 cells with PAUPAR overexpression, and anti-miR-17-5p was transfected into HCT116 cell with PAUPAR knockdown (Figure 5(a)). The up-regulation of miR-17-5p was validated to attenuate the inhibitory effect of PAUPAR overexpression on the malignant phenotype of HCT116 cells (Figure 5(b–f)). Moreover, the oncogenic effects of knocking down PAUPAR on HCT8 cells was partially reversed by anti-miR-17-5p (Figure 5(b–f)). Additionally, the Western blot implied that ZNF750 expression was repressed after PAUPAR knockdown in HCT116 cells, and this effect was partially attenuated by miR-17-5p inhibition; on the other hand, ZNF750 expression was increased by PAUPAR overexpression in HCT8 cells, and this effect was counteracted by miR-17-5p overexpression (Figure 5(g)). These data suggested that PAUPAR promoted the expression of ZNF750 via repressing miR-17-5p.

(a) HCT8 cells with PAUPAR overexpression were transfected with miR-17-5p mimics; HCT116 cells with PAUPAR knockdown were transfected with anti-miR-17-5p. Then, miR-17-5p expression in CRC cells was determined by qRT-PCR. (b,c) Colony formation assay and BrdU assay were used to detect cell proliferation. (d,e) Transwell experiments were used to detect cell migration and invasion. (f) The apoptosis of CRC cells was detected by TUNEL assay. (g) Western blot was used to detect ZNF750 expression in HCT116 and HCT8 cells. All the experiments were performed in triplicate. ** *P* < 0.01, *** *P* < 0.001.

### PAUPAR represses the metastatic potential of HCT8 cells in vivo

**Figure 6.**
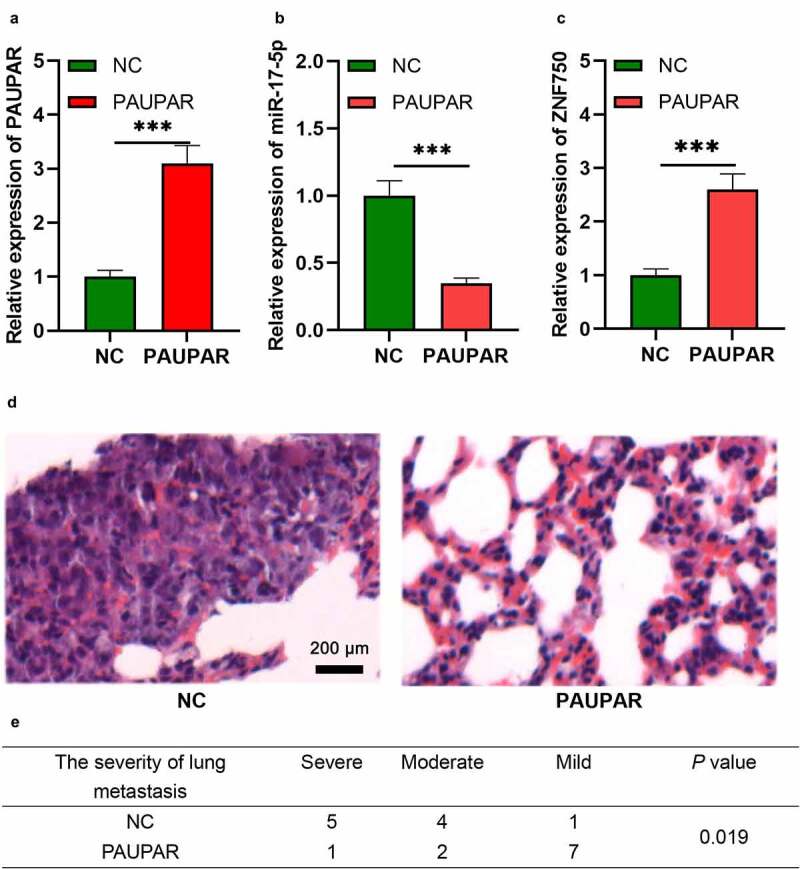
PAUPAR repressed tumor metastasis *in vivo.*

To further validate the role of PAUPAR *in vivo*, we constructed a lung metastasis model of HCT8 cells with nude mice. As shown, after PAUPAR overexpression plasmids were transfected into HCT8 cells, the expression of PAUPAR and ZNF750 was up-regulated, while the expression of miR-17-5p was down-regulated (Figure 6(a–c)). The cells and the control cells were then injected into nude mice via the caudal vein. Two weeks later the mice were sacrificed,, and the lung tissues were harvested. Next, HE staining was performed to measure the lung metastasis of HCT8 cells. The pathological examination suggested that PAUPAR markedly reduced the severity of lung metastasis of HCT8 cells (Figure 6(d,e)). These results further indicate that PAUPAR could restrict the malignant biological behavior of CRC cells.

(a-c) qRT-PCR was employed to detect the expression of PAUPAR, miR-17-5p, and ZNF750 after HCT8 cells were transfected with PAUPAR overexpression plasmids. (d,e) HE staining was employed to detect the lung metastasis of the mice.*** *P* < 0.001.

## Discussion

Accumulating research has demonstrated that many lncRNAs are aberrantly expressed in CRC and they modulate the malignant phenotypes of tumor cells [26]. Further investigation of the biological function of lncRNAs in CRC may bring new therapeutic targets for CRC patients. PAUPAR exerts multiple biological effects, such as accelerating the differentiation and development of pancreatic α-cells in the pancreas [27]. In the central nervous system, PAUPAR can bind to KRAB domain-associated protein 1 to modulate neuronal differentiation [14]. It has also been reported that PAUPAR overexpression in the nervous system remarkably facilitates c-Jun-N-terminal kinase (JNK) and p-JNK expression and enhances apoptosis [28]. This work reveals that PAUPAR expression in CRC tissues is markedly reduced, and its low expression is remarkably associated with adverse clinical parameters in CRC patients, and these results indicate that PAUPAR has the potential to become a biomarker for CRC’s diagnosis and prognosis prediction. Furthermore, functional experiments validate that PAUPAR overexpression markedly represses the proliferation, migration, and invasion of CRC cells relative to controls, while knocking down PAUPAR enhances the above malignant biological behavior of CRC cells. This study implies that PAUPAR is tumor-suppressive in CRC.

MiRNAs are extensively involved in carcinogenesis and cancer progression. Many studies have verified that miR-17-5p can modulate the malignant phenotype of tumor cells. For instance, miR-17-5p enhances the proliferation and metastasis of cervical cancer cells via targeting the transforming growth factor β receptor 2 [29]. In osteosarcoma cells, miR-17-5p enhances osteosarcoma cell proliferation and EMT via targeting the SRC kinase signaling inhibitor 1 [30]. These demonstrations suggest that miR-17-5p exerts a pro-cancer effect in tumors. The present work also reveals that miR-17-5p is overexpressed in CRC and exerts a pro-cancer effect, which is consistent with the previous report [21]. Additionally, dual-luciferase reporter assay, RIP assay, and qRT-PCR prove that PAUPAR adsorbs miR-17-5p and negatively modulates the latter’s expression. Rescue experiments reveal that miR-17-5p partially reverses the cancer-suppressive effect of PAUPAR. Hence, we conclude that the PAUPAR/miR-17-5p axis is implicated in regulating CRC progression.

ZNF750 can participate in the terminal differentiation of epithelial cells through the activation of kruppel-like factor 4, which can impede cell proliferation and promote transcription of differentiation-associated genes [31]. ZNF750 is reported to exhibit tumor-suppressive effects in esophageal squamous cell carcinoma and oral squamous cell carcinoma [32,33]. Mechanistically, ZNF750 suppresses the expression of some cancer-related genes such as angiogenin, VEGF, RGS5, CD105, ITGA5, ITGB1, and CD44 [32]. Notably, as a crucial protein negatively regulating the G protein signaling pathway, RGS5 is highly expressed in perivascular cells of diverse tumor tissues and is crucial in angiogenesis and vascular remodeling during cancer progression, and it reduces the efficacy of tumor radiotherapy, chemotherapy, and immunotherapy [34,35]. This suggests that ZNF750 may be a promising target for sensitizing cancer cells to therapy. A previous research has shown that miR-17-5p targets 3ʹ UTR of ZNF750 and modulates the expression of ZNF750 to promote CRC cells’ proliferation, migration, and invasion [21]. In the present work, we observed that PAUPAR could up-regulate the expression of ZNF750 via repressing miR-17-5p. Our data imply that ZNF750 may be a crucial downstream mediator by which PAUPAR/miR-17-5p axis participates in regulating CRC progression.

## Conclusion

Taken together, this work demonstrates that PAUPAR expression is down-modulated in CRC and PAUPAR can represses the malignancy of CRC cells through the miR-17-5p/ZNF750 axis. The findings of this work provide a novel theoretical basis for the diagnosis and therapy of CRC.

## Supplementary Material

Supplemental Material

## Data Availability

The data used to support the findings of this study are available from the corresponding author upon request.

## Highlights

- PAUPAR is markedly down-modulated in CRC, and its low expression is significantly related to the unfavorable clinicopathological characteristics.
- Knockdown of PAUPAR promotes CRC cells’ proliferation, migration and invasion, and represses apoptosis.
- PAUPAR impedes CRC malignant biological features via modulating the miR-17-5p/ZNF750 axis.
