# Supplementary material for: PAX6 upstream antisense RNA (PAUPAR) inhibits colorectal cancer progression through modulation of the microRNA (miR)-17-5p / zinc finger protein 750 (ZNF750) axis
Source: Bioengineered. 2021 Jul 21;12(1):3886–99. doi: 10.1080/21655979.2021.1940071 (PMC8806802; doi:10.1080/21655979.2021.1940071)
Supplement: Supplemental Material [file KBIE_A_1940071_SM3647.zip › Supplementary figure caption.docx]

**Supplementary figure 1**

1. 3 online databases (miRDIP, TargetScan, and StarBase ) were searched computationally for potential target genes that are targeted by miR-17-5p.

(B & C) KEGG pathway analysis and GO analysis were performed to predict the potential biological functions of miR-17-5p integrated-signature

**Supplementary figure 2 Apoptosis of CRC cells was detected by TUNEL assay, and the representative images were shown.**
