# Supplementary figures and images for: PAX6 upstream antisense RNA (PAUPAR) inhibits colorectal cancer progression through modulation of the microRNA (miR)-17-5p / zinc finger protein 750 (ZNF750) axis
Source: Bioengineered. 2021 Jul 21;12(1):3886–99. doi: 10.1080/21655979.2021.1940071 (PMC8806802; doi:10.1080/21655979.2021.1940071)

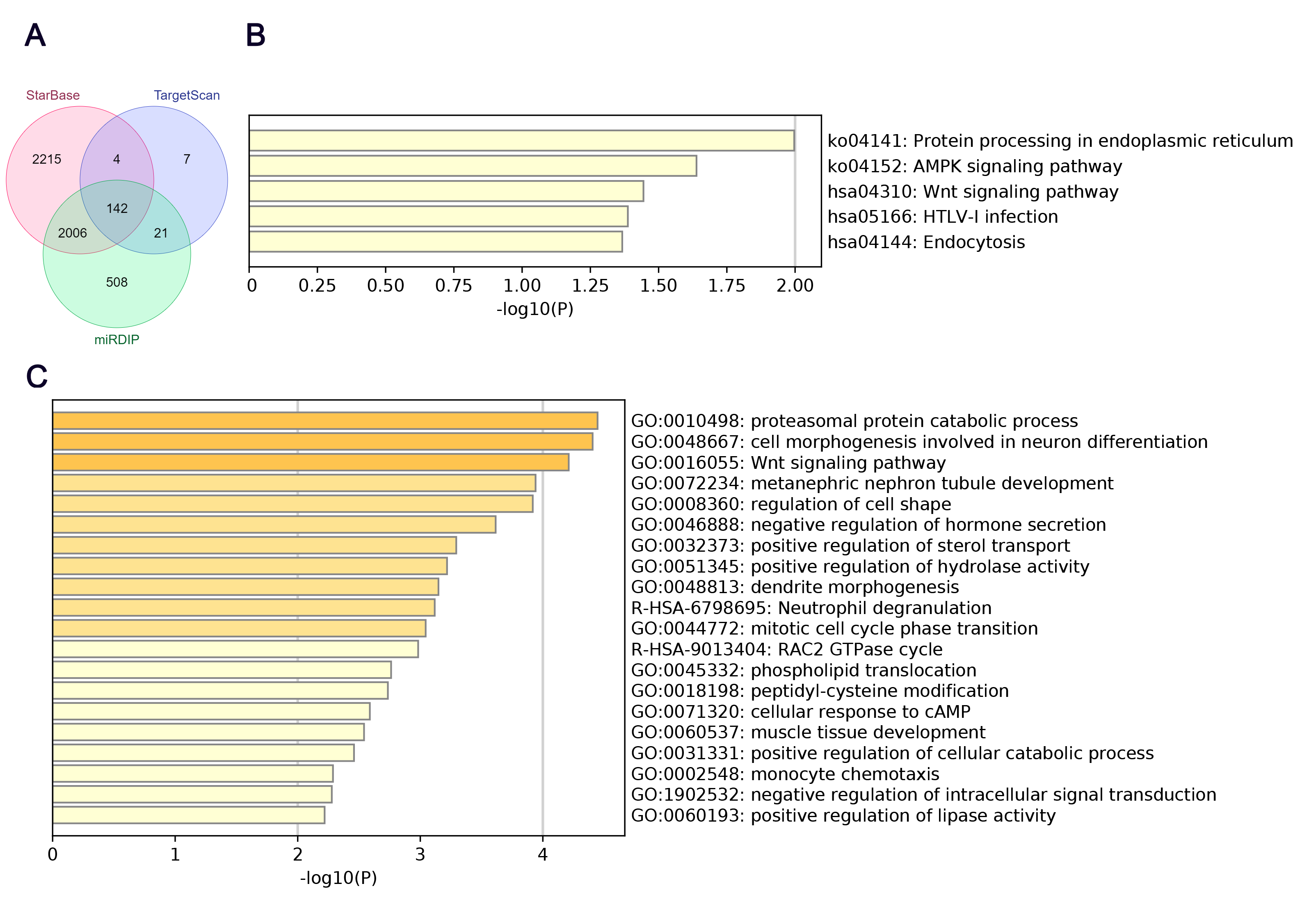

Supplement: Supplemental Material [file KBIE_A_1940071_SM3647.zip › Supplementary figure 1.tif]

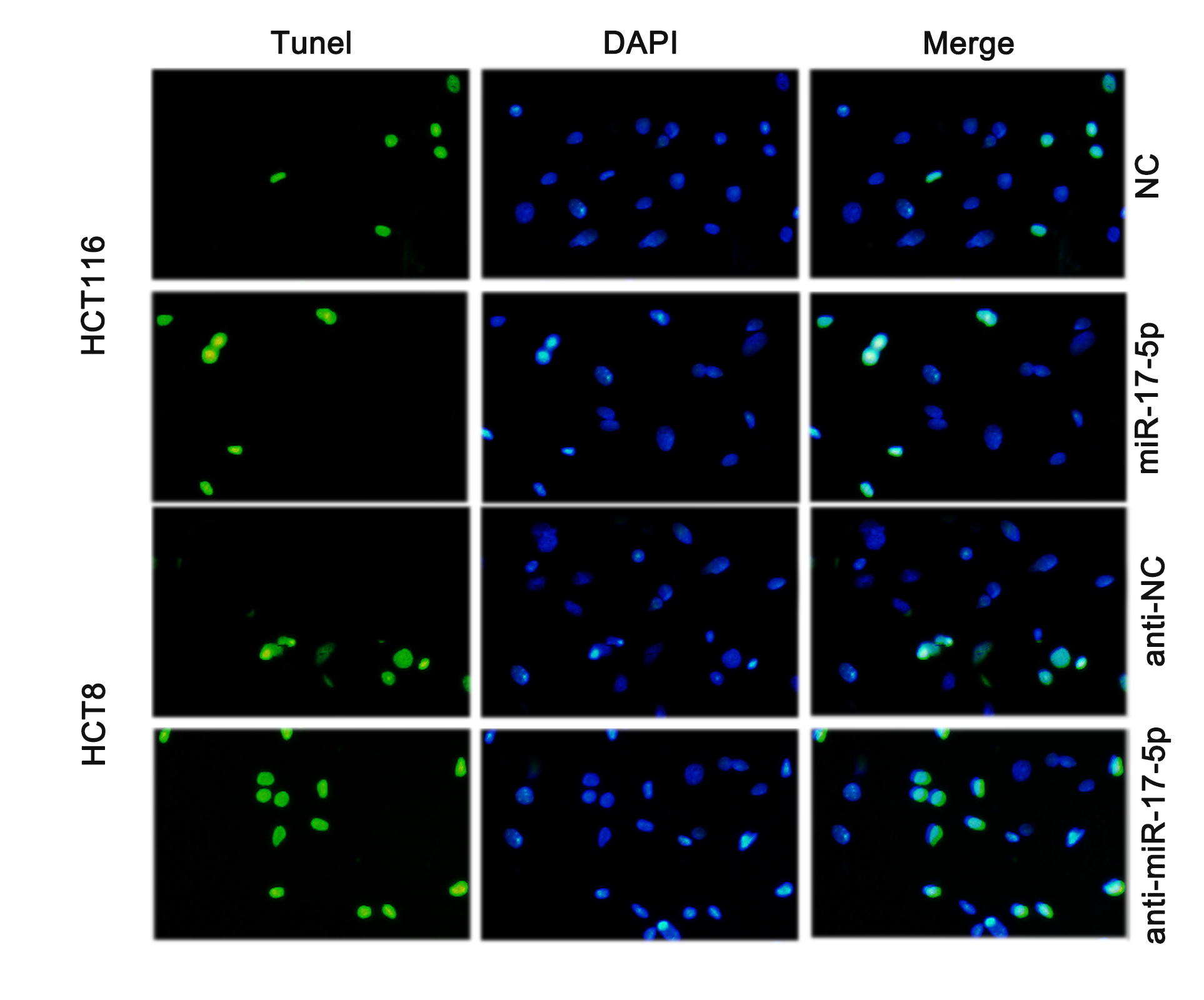

Supplement: Supplemental Material [file KBIE_A_1940071_SM3647.zip › Supplementary figure 2.tif]
